# Artificial intelligence-assisted diagnosis of ocular surface diseases

**DOI:** 10.3389/fcell.2023.1133680

**Published:** 2023-02-17

**Authors:** Zuhui Zhang, Ying Wang, Hongzhen Zhang, Arzigul Samusak, Huimin Rao, Chun Xiao, Muhetaer Abula, Qixin Cao, Qi Dai

**Affiliations:** ^1^ The First People’s Hospital of Aksu District in Xinjiang, Aksu City, China; ^2^ National Clinical Research Center for Ocular Diseases, Eye Hospital, Wenzhou Medical University, Wenzhou, China; ^3^ Huzhou Traditional Chinese Medicine Hospital Affiliated to Zhejiang University of Traditional Chinese Medicine, Huzhou, China

**Keywords:** artificial intelligence, deep learning, machine learning, ocular surface diseases, convolutional neural network

## Abstract

With the rapid development of computer technology, the application of artificial intelligence (AI) in ophthalmology research has gained prominence in modern medicine. Artificial intelligence-related research in ophthalmology previously focused on the screening and diagnosis of fundus diseases, particularly diabetic retinopathy, age-related macular degeneration, and glaucoma. Since fundus images are relatively fixed, their standards are easy to unify. Artificial intelligence research related to ocular surface diseases has also increased. The main issue with research on ocular surface diseases is that the images involved are complex, with many modalities. Therefore, this review aims to summarize current artificial intelligence research and technologies used to diagnose ocular surface diseases such as pterygium, keratoconus, infectious keratitis, and dry eye to identify mature artificial intelligence models that are suitable for research of ocular surface diseases and potential algorithms that may be used in the future.

## 1 Introduction

Artificial intelligence (AI) is a frontier field of computer science whose goal is to use computers to solve practical issues (Rahimy, 2018). The concept was introduced at a workshop at Dartmouth College in 1956 (Lawrence et al., 2016). The conference discussed the relevant theories and principles of machine simulation intelligence. Since then, the development of AI has been unstable due to limited technical conditions and levels. Nevertheless, with the rapid development of computer technology, the application of AI in medical research has become a hot topic in modern technology. Recently, healthcare has become one of the frontiers of AI applications, particularly for image-centric subspecialties such as ophthalmology (Ting et al., 2019), cardiology (Dey et al., 2019), radiology (Saba et al., 2019), and oncology (Niazi et al., 2019), among others. They adopt big data technology to collect massive clinical data and images and apply big medical data to AI to guide or assist doctors in clinical decision-making through the supercomputing power and data mining ability of cloud computing. AI can obtain disease characteristics from the training set and apply them to a verification or test set to diagnose the corresponding disease. AI can segment anatomical structures such as abnormal shapes in the images. AI can also classify images into different types according to the characteristics of diseases. The algorithms of AI include traditional machine learning (ML) algorithm and deep learning (DL) algorithm. The traditional ML algorithms mainly include linear regression, logical regression, support vector machine (SVM), decision tree and random forest (RF) algorithms, and usually do not involve large-scale neural networks. DL algorithm mainly uses multimedia data sets (such as images, videos, and sounds), and usually involves the application of large-scale neural networks, including artificial neural network (ANN), convolutional neural network (CNN), and recurrent neural network (RNN).

Previously, most studies on the application of AI in ophthalmology focused on glaucoma (Devalla et al., 2018; Kucur et al., 2018; Asaoka et al., 2019; Wang M. et al., 2019), fundus diseases (Gulshan et al., 2016; Burlina et al., 2017; Ting et al., 2017; Venhuizen et al., 2018; Nagasato et al., 2019), and cataracts (Gao et al., 2015; Yang et al., 2016; Long et al., 2017; Wu et al., 2019; Xu et al., 2020). Compared to diagnosing retinal diseases, which largely depend on fundus images acquired from ophthalmoscopy or fundus photography, multiple examinations are required to diagnose ocular surface diseases, considering the complexity of their structural and physiological functions. In recent years, with the expansion of AI in ophthalmology, increasing research has applied AI to ocular surface diseases such as pterygium, keratoconus (KC), infection keratitis, and dry eye. Herein, we reviewed research on the application of AI in the field of ocular surface-related diseases to guide clinical work. The remainder of this paper consists of the following: Sections 2–7 provides the efficiency of AI in diagnosing ocular surface diseases, pterygium, KC, infectious keratitis, dry eye, and other ocular surface diseases.

**FIGURE 1:**
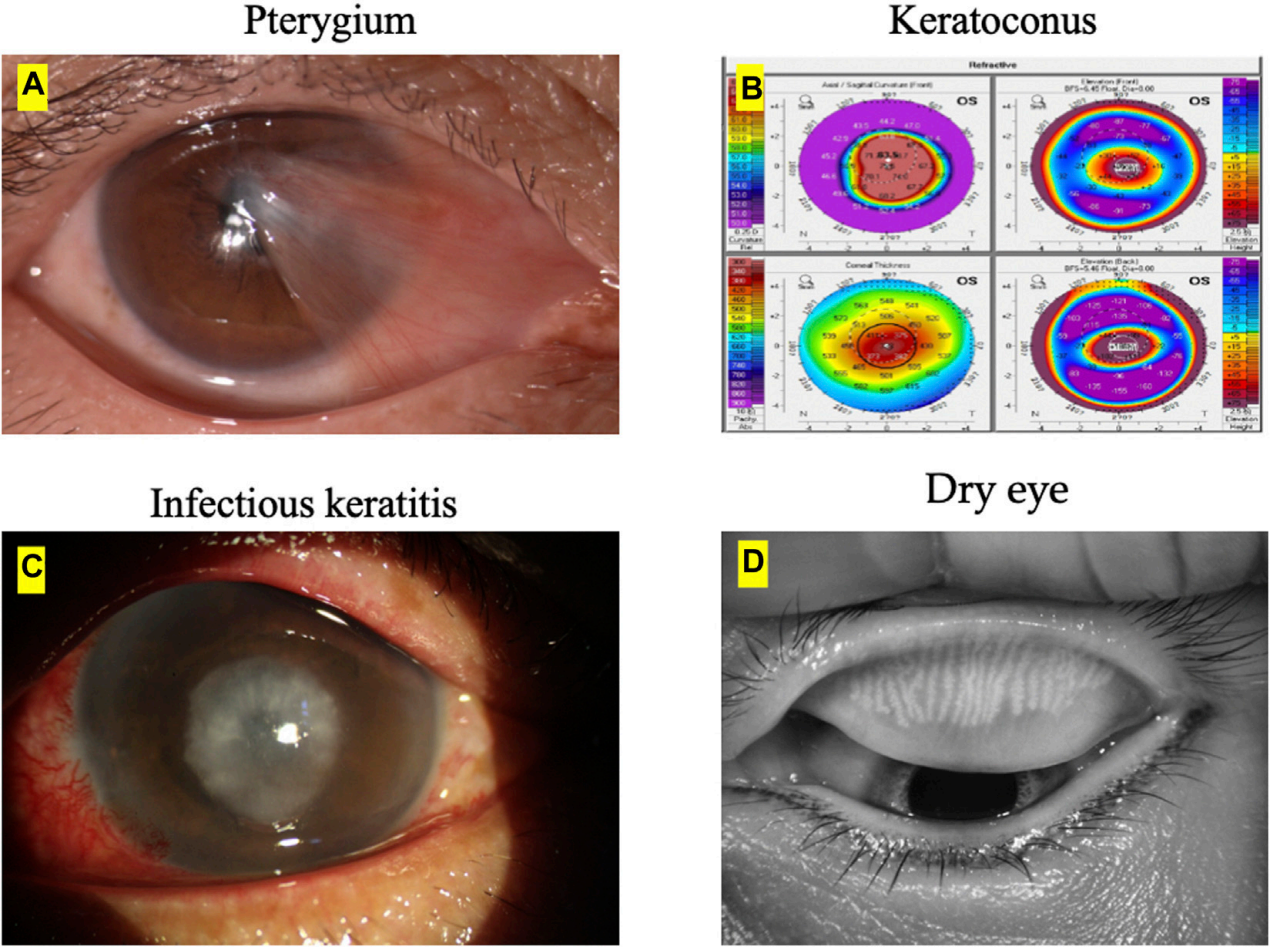
Ocular surface diseases and image modalities. **(A)** The main imaging modality of pterygium is anterior segment photograph. **(B)** The main imaging modality of keratoconus is pentacam. **(C)** The main imaging modality of infectious keratitis is slit-lamp images. **(D)** The main imaging modality of dry eye is Keratograph 5M.

The image examples of ocular surface diseases and image modalities to diagnose each corneal disease is presented in Figure 1. The main image modalities of ocular surface diseases include anterior segment photograph, pentacam, slit-lamp images and Keratograph 5M, etc.

## 2 Search methods

A systematic literature search was performed in PubMed and Web of science. The goal was to retrieve as many studies as possible applying ML to ocular surface disease related data. The following keywords were used: All combinations of “ocular surface,” “pterygium,” “keratoconus,” “keratitis,” “dry eye,” and “meibomian gland dysfunction (MGD)” with “artificial intelligence,” “machine learning,” “deep learning,” “convolutional neural network,” “decision tree.” No time period limitations were applied for any of the searches.

## 3 AI application in pterygium

Pterygium is a common eye disorder in which abnormal fibrovascular tissue protrudes from the inner side of the eyes toward the corneal area (Zulkifley et al., 2019). Since it is directly linked to excessive exposure to ultraviolet radiation, farmers and fishermen are the two high-risk groups (Gazzard et al., 2002; Abdani et al., 2019). This condition can be better managed when patients know about this disease early. Moreover, pterygium tissues or lesions encroach on the pupil area at the latter stage, possibly causing vision impairment (Tomidokoro et al., 2000; Clearfield et al., 2016; Wang F. et al., 2021). Currently, the grading of pterygium is mainly based on the subjective evaluation of doctors. Therefore, AI can be used to develop an efficient automatic grading system for pterygium (Hung et al., 2022). In vast rural and remote areas that lack professional medical resources for ophthalmology, AI diagnostic technology can provide local patients with a convenient pterygium screening method, prevent the rush of patients to county or prefectural hospitals for medical care, and reduce the burden on patients. Furthermore, it suggests treatment methods, clarifies the indications for further surgical treatment, facilitates the timely referral of patients needing surgery at the grassroots level, and rationally allocates medical resources. Table 1 mainly reviews AI applications for the diagnosis of pterygium.

**TABLE 1:** Summary of studies focused on computer-aided pterygium diagnosis.

Year	Authors	Imaging modality	Image size	Databases	AI algorithms	AUC (%)	Accuracy (%)	Sensitivity (%)	Specificity (%)	IoU (%)
2018	Wan Zaki et al. (2018)	ASP	3,017	Normal and pterygium	SVM/ANN	95.60	91.27	88.70	88.30	—
2018	Zhang et al. (2018)	ASP	1,513	Normal, pterygium, keratitis, subconjunctival hemorrhage, and cataract	CNN/Faster-RCNN	95.95	>95.00	97.45	71.15	—
2019	Zulkifley et al. (2019)	ASP	120	Normal and pterygium	FCNN	97.0	81.10	95.0	98.3	—
2020	Abdani et al. (2020)	ASP	328	Ranging from early to late stage of pterygium	CNN	—	92.02	—	—	92.02
2021	Xu W. et al. (2021)	ASP	1,220	Normal, observation (pterygium) and operation (pterygium)	DL (EfficientNet-B6)	>93.00	94.68	>90.00	>95.00	—
2021	Abdani et al. (2021)	ASP	328	Ranging from early to late stage of pterygium	CNN	—	93.30	—	—	86.40
2021	Fang et al. (2022)	ASP	9,443	Pterygium and referable pterygium	CNN	≥98.50	≥95.2	≥94.0	≥95.30	—
2022	Hung et al. (2022)	SLI	237	Normal, primary and recurrent pterygium	DL	—	80.00	66.67	81.82	—
2022	Wan et al. (2022)	ASP	489	Normal, observation (pterygium) and operation (pterygium)	DL(U-Net++)	95.86	92.37	90.24	93.51	>86.40

AUC, area under the curve; IoU, intersection over union; ASP, anterior segment photograph; SVM, support vector machine; ANN, artificial neural networks; CNN, convolutional neural network; Faster-RCNN, faster-region based convolutional neural network; FCNN, fully convolutional neural networks; DL, deep learning; SLI, slit-lamp images.

In 2012, Gao et al. (2012) proposed a pterygium detection system based on color information. Interestingly, the pupil detection technique, which uses corneal images, achieved 85.38% accuracy. Similarly, Mesquita and Figueiredo (2012) applied a circle hough transform to segment the iris. Subsequently, a region-growing algorithm based on Otsu’s algorithm is applied to the iris’s segmented area to segment the pterygium tissue. Wan Zaki et al. (2018) developed an image-processing method based on ASP using the following four modules to differentiate pterygium from normal: preprocessing, corneal segmentation, feature extraction, and classification. Image-processing method performance was evaluated using a SVM and an ANN. The performance of the proposed image-processing method generated results of 88.7%, 88.3%, and 95.6% for sensitivity, specificity, and area under the curve (AUC), respectively. However, the imperfect image setup should also be noted as a limitation. Abdani et al. (2020) and Abdani et al. (2021) proposed an automatic pterygium tissue segmentation using CNN. This is useful for detecting pterygium from the early stage to the late stage. The overall accuracy of both studies is high [92.20% (Abdani et al., 2020), 93.30% (Abdani et al., 2021)].

**FIGURE 2:**
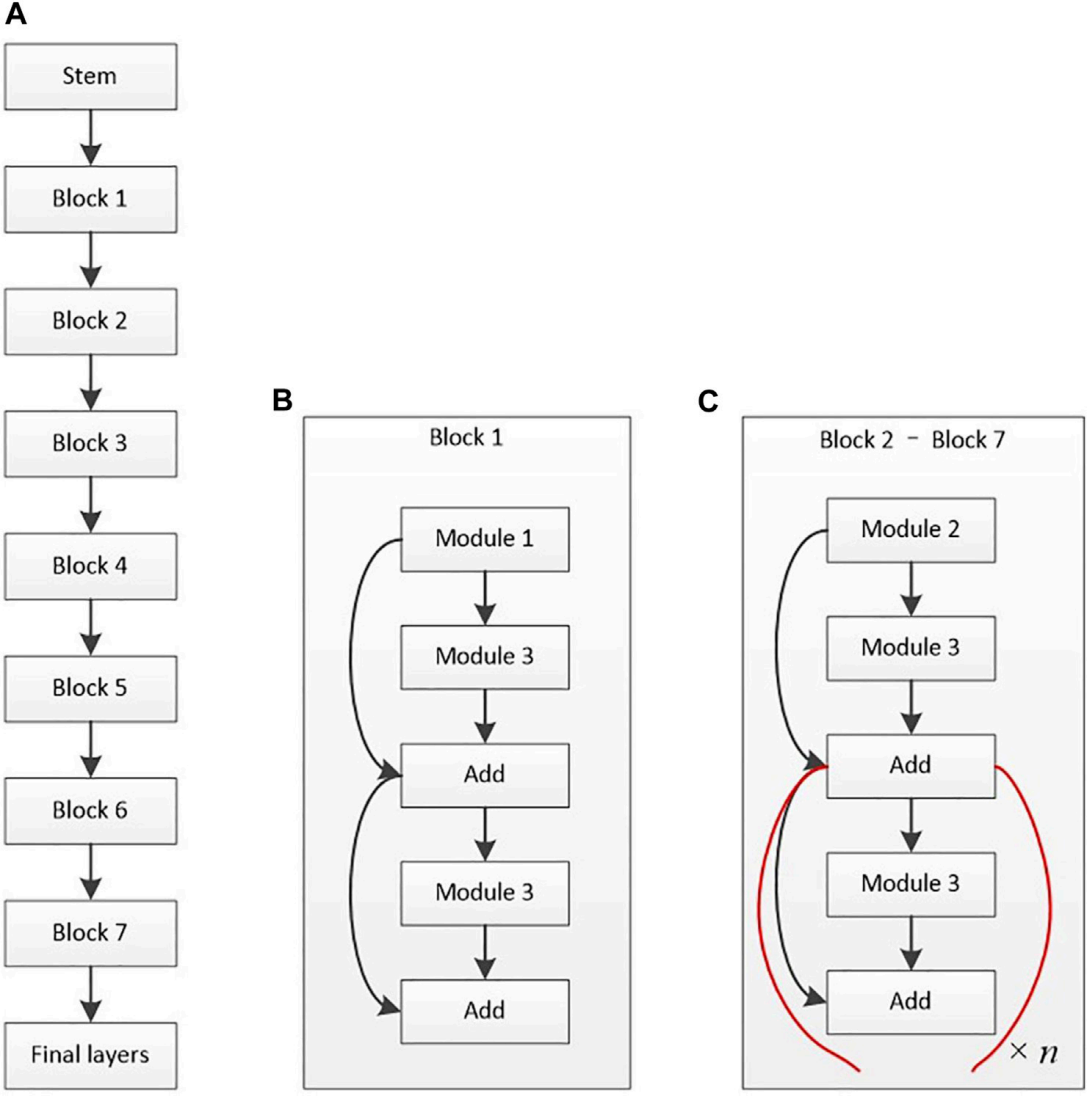
Architectural diagram of EfficientNet-B6 (Xu W. et al., 2021). **(A)** Basic architecture. **(B)** Structure of block 1. **(C)** Structure of blocks 2–7.

Zhang et al. (2018) also used a deep DL diagnosis system that can automatically diagnose various eye diseases based on the patient’s ASP and provide diagnosis-based targeted treatment recommendations. Specifically, the last stage provides treatment advice based on medical experience and AI strictly associated with pterygium (accuracy, >95%). Zulkifley et al. (2019) proposed a DL approach (Pterygium-Net) based on fully convolutional neural networks (FCNN) with the help of transfer learning to detect and localize the pterygium automatically. Pterygium-Net produces high average detection sensitivity and specificity of 0.95 and 0.983, respectively. As for pterygium tissue localization, the algorithm achieves 0.811 accuracies with a meager failure rate of 0.053. Xu W. et al. (2021) developed a unique intelligent diagnosis system based on DL to diagnose pterygium (Figure 2 depicts the architectural diagram of EfficientNet-B6, created by Xu et al.). Experts and the AI diagnosis system categorized the images into the following three categories: normal, pterygium observation, and pterygium surgery. Moreover, the accuracy rate of the AI diagnostic system on the 470 tested images was 94.68%, diagnostic consistency was high, and kappa values of the three groups were above 85%. The AI, pterygium diagnosis system, can not only judge the presence of pterygium but also classify the severity of pterygium. Fang et al. (2022) evaluated the performance of a DL algorithm for the detection of the presence and extent of pterygium based on ASP taken from slit-lamp and handheld cameras. The AI algorithm could detect the presence of referable-level pterygium with optimal sensitivity and specificity. A handheld camera might be a simple screening tool for detecting reference pterygium.

Hung et al. (2022) proposed a DL system to predict pterygium recurrence. The AI algorithm shows high specificity (80.00%) but low sensitivity (66.67%) in predicting pterygium recurrence. Wan et al. (2022) proposed a DL system for measuring the pathological progression of pterygium. These are essential for achieving accurate medical diagnosis and can conveniently assist ophthalmologists in timely detecting pterygium status and arranging surgery strategies. In addition to the abovementioned application of AI to the segmentation and diagnosis of pterygium, Kim et al. (2022) developed AI software for quantitative analysis of the immunochemical image of pterygium. They concluded that the AI software might improve the reliability and accuracy of evaluating histopathological specimens obtained after ophthalmological surgery. The above research shows that the AI model can achieve satisfactory results in the diagnosis and classification prediction of pterygium.

## 4 AI application in KC

KC is a non-inflammatory, asymmetric, ectatic corneal disorder characterized by progressive thinning and impaired vision (Henein and Nanavaty, 2017; Mas Tur et al., 2017). Since the signs of intermediate and advanced KC are quite common, clinical diagnosis is straightforward (Gomes et al., 2015). Atypical KC includes KC suspect (KCS), forme fruste KC (FFKC), and subclinical KC (SKC). Unfortunately, these atypical KC symptoms and signs are not obvious and are difficult to diagnose based on general examination results. However, most of the KC studies analyzed the corneal morphological metrics from Pentacam. AI-based corneal morphological metrics can provide early KC detection. Moreover, early AI research on KC relied on corneal topography data for neural network training to distinguish KC from other corneal abnormalities such as astigmatism, corneal transplantation, and post-photorefractive keratectomy (PRK). Table 2 mainly reviews AI applications for the diagnosis of KC.

**TABLE 2:** Summary of studies focused on computer-aided KC diagnosis.

Year	Authors	Imaging modality	Image size	Databases	AI algorithms	AUC (%)	Accuracy (%)	Sensitivity (%)	Specificity (%)	IoU (%)
1997	Smolek and Klyce (1997)	TMS-1	300	KC, KCS, and others	CNN	—	100.00	100.00	100.00	—
2002	Accardo and Pensiero (2002)	EyeSys	396	Normal, KC, and others	CNN	—	96.70	94.10	97.60	—
2005	Twa et al. (2005)	Keratron	244	Normal and KC	MLC	97.00	92.00	92.00	93.00	—
2010	Souza et al. (2010)	Orbscan II	318	Normal, astigmatism, KC, and PRK	SVM/MLP/RBFNN	>98.00	—	100.00	98.00	—
2012	Arbelaez et al. (2012)	Sirius	3,502	Normal, SKC, KC and PRK	SVM	—	98.20	95.00	99.30	—
2013	Smadja et al. (2013)	Galilei	372	Normal, FFKC, and KC	MLC	—	—	99.50	100.00	—
2016	Ruiz Hidalgo et al. (2016)	Pentacam	860	Normal, astigmatism, FFKC, KC, and PRK	SVM/MLC	99.80	98.90	99.10	98.50	—
2016	Kovács et al. (2016)	Pentacam	135	Normal fellow eyes with unilateral KC, bilateral KC, and PRK	CNN/MLC	99.00	—	100.00	95.00	—
2018	Yousefi et al. (2018)	CASIA AS-OCT	3,156	Normal, FFKC, mild KC, and advanced KC	Unsupervised ML	—	—	97.70	94.10	—
2019	Zou et al. (2019)	Pentacam	2018	Normal, SKC, and KC	SVM-RFE/GBDT	99.82	98.91	76.92	100	—
2019	Dos Santos et al. (2019)	UHR-OCT	20,160	Normal and KC	CNN (CorneaNet)	—	99.56	>99.30	—	>98.50
2019	Issarti et al. (2019)	Pentacam	838	Normal and KCS and mild-moderate KC	FFN	—	96.56	97.78	95.56	—
2019	Kamiya et al. (2019)	CASIA AS-OCT	304	Normal and grade 1–4 KC	CNN	—	99.10	100	98.40	—
2019	Lavric and Valentin (2019)	SyntEyes	400	Normal and KC	CNN	—	99.33	—	—	—
2020	Kuo B. I et al. (2020)	TMS-4+ Pentacam + Corvis ST	354	Normal, SKC, and KC	CNN	99.50	95.80	94.40	97.20	—
2020	Abdelmotaal et al. (2020)	Pentacam	3,218	Normal, SKC, and KC	CNN/SVM	—	>95.50	>92.00	>92.00	—
2020	Shi et al. (2020)	Pentacam + UHR-OCT	121	Normal, SKC, and KC	MLC	100	—	100	100	—
2020	Xie et al. (2020)	Pentacam	6,465	Normal, suspected irregular cornea, early KC, KC, and PRK	CNN/TL	99.90	95.0	97.80	99.20	—
2020	Cao et al. (2020)	Pentacam	88	Normal and SKC	RF	96.00	87.00	88.00	85.00	—
2021	Cao et al. (2021)	Pentacam	267	Normal and SKC	RF	—	98.00	97.00	98.00	—
2021	Al-Timemy et al. (2021)	Pentacam	3,794	Normal, KCS, and KC	CNN/SVM	99.00	97.70	—	—	—
2021	Herber et al. (2021)	Pentacam + Corvis ST	434	Normal, mild, moderate, and advanced KC eyes	RF	97.00	93.00	91.00	94.00	—
2021	Castro-Luna et al. (2021)	Pentacam + Corvis ST	81	Normal and SKC	RF	—	89.00	86.00	93.00	—
2021	Kamiya et al. (2021a)	TMS-4	349	Normal and grade 1–4 KC	CNN	99.70	96.60	98.80	94.40	—
2021	Chen et al. (2021)	Pentacam	1926	Normal and grade 1–4 KC	CNN	94.23	97.85	98.46	90.00	—
2021	Malyugin et al. (2021)	Pentacam	800	Normal and grade 1–4 KC	ML/QDA	97.00	97.00	—	—	—
2021	Ghaderi et al. (2021)	Pentacam	450	Normal and grade 1–3 KC	MLP/ANFIS	—	98.20	99.10	96.20	—
2021	Kamiya et al. (2021b)	CASIA AS-OCT	218	Non-progressive and progressive KC	CNN	—	84.90	95.50	58.10	—
2021	Kato et al. (2021)	Pentacam	274	Non-progressive and progressive KC	CNN	81.40	—	77.80	69.60	—
2021	Kundu et al. (2021)	MS-39	1,122	Normal, VAE, and KC	RF/ZP	99.70	99.10	98.70	—	—
2021	Aatila et al. (2021)	CASIA AS-OCT	12, 242	Normal, FFKC, mild KC, and advanced KC	RF	100	98.00	98.00	—	—
2022	Mohammadpour et al. (2022)	Pentacam, Sirius and OPD-Scan III	212	Normal, SKC, and KC	MLC	—	91.24	80.00	96.60	—
2022	Tan et al. (2022)	Corvis ST	354	Normal and KC	FFN	—	99.60	99.30	100	—
2022	Ahn et al. (2022)	Pentacam	1,246	Normal, SKC, and KC	Ensemble	—	85.40	96.40	83.10	—
2022	Xu et al. (2022)	Pentacam	1,108	Normal, AKC, and KC	CNN	100	98.77	98.48	98.96	—

KC, keratoconus; KCS, keratoconus suspect; MLC, machine learning classification; PRK, photorefractive keratectomy; MLP, multi-layer perceptron; RBFNN, radial basis function neural network; SKC, subclinical keratoconus; FFKC, forme fruste keratoconus; AS-OCT, anterior segment optical coherence tomography; ML, machine learning; RFE, recursive feature elimination; GBDT, gradient boosting decision tree; UHR-OCT, ultra-high-resolution optical coherence tomography; FFN, feedforward neural network; TL, transfer learning; RF, random forest; QDA, quadratic discriminant analysis; ANFIS, adaptive network-based fuzzy inference system; VAE, very asymmetric ectasia; ZP, zernike polynomials; AKC, asymmetric keratoconus. TMS: A computer-assisted videokeratoscope (Tomey Corporation, Nagoya, Japan). MS-39: A hybrid tomographer (CSO, Florence, Italy).

**FIGURE 3:**
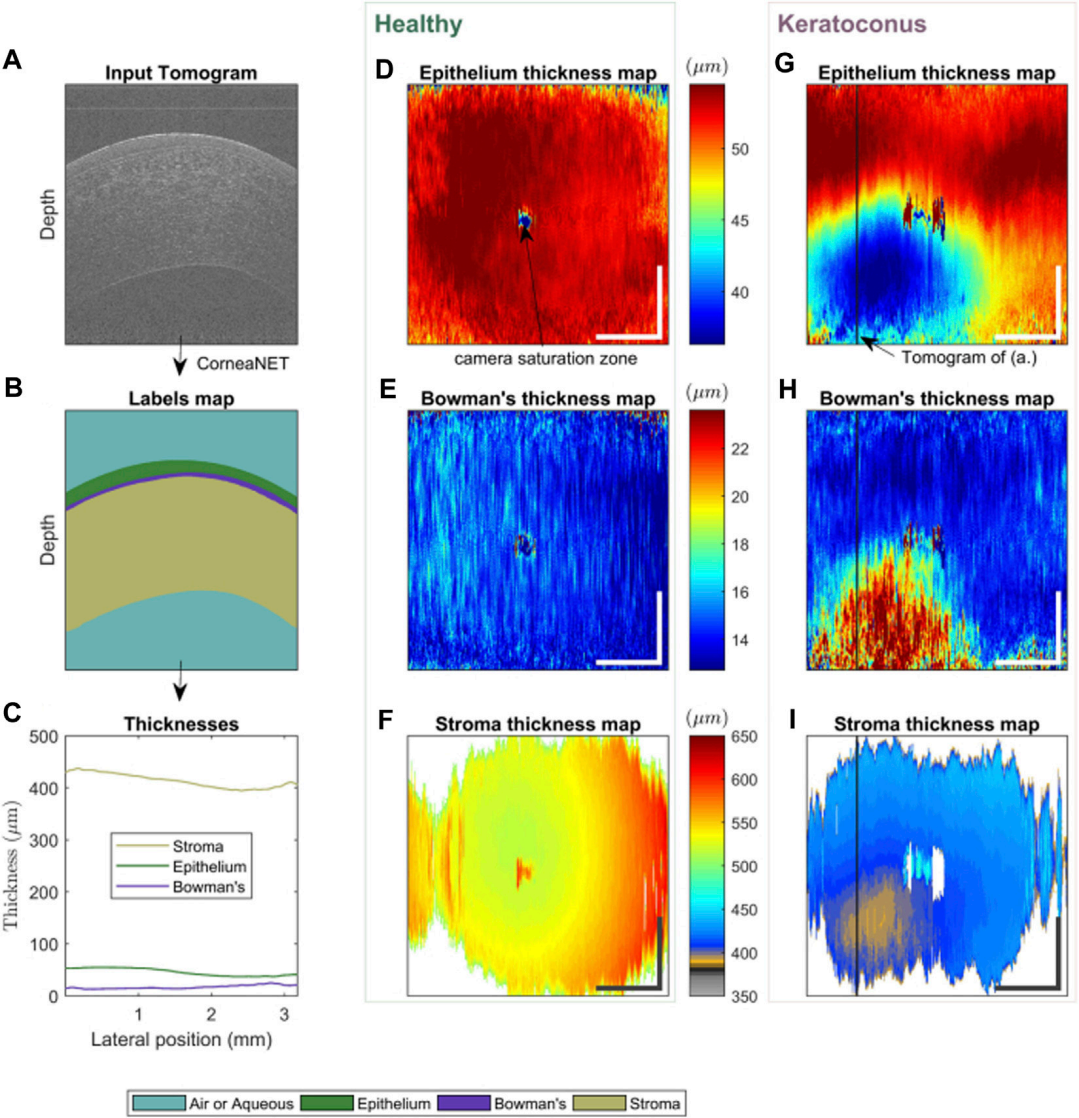
Using CorneaNet, the thicknesses of the epithelium, stroma, and Bowman’s layer were computed in a normal and a KC case (Dos Santos et al., 2019). The healthy case shows close to uniform thicknesses for all three layers. In contrast, for the KC case, the epithelium and stroma are thinner in a specific region of the cornea, and Bowman’s layer is thicker. **(A–C)** Thickness calculation in one tomogram. **(A)** UHR-OCT tomogram of a keratoconus patient, **(B)** corresponding labels map computed using CorneaNet. **(B)** Thicknesses of the three corneal layers computed using the label maps. **(D–F)** Thickness maps in a healthy subject case. **(G–I)** Thickness maps in a keratoconus case. The thickness scale bar is shared by the maps horizontally. Scale bar: 1 mm.

The advantage of these AI algorithms lies in the potential to help clinicians differentiate between KC and normal eyes. In 1997, Smolek and Klyce (1997) designed a classification neural network for KC screening to detect the existence of KC or KCS. In total, 10 topographic indices were used as the network inputs. The AI model showed 100% accuracy, specificity, and sensitivity for the test set. Accardo and Pensiero (2002) proposed an ANN method to identify KC from corneal topographies. The results showed a global sensitivity and specificity of 94.1% (with a KC sensitivity of 100%) and 97.6% (98.6% for KC alone) in the test set, respectively. This study elevates the potential of AI for the automatic screening of early KC, pointing out that simultaneously using the topographic parameters of both eyes improves the discriminative capability of the ANN. Twa et al. (2005) described applying decision tree induction, an automated machine learning classification (MLC) approach, to objectively and quantitatively differentiate between normal and KC corneal shapes. The results showed an accuracy of 92% and an area under the receiver operating characteristic (ROC) curve of 0.97. Arbelaez et al. (2012) employed the SVM algorithm to integrate data from the corneal surfaces and pachymetry into the model. Interestingly, precision was improved the most when posterior corneal surface data were included, particularly in SKC cases. Additionally, this AI approach increases its sensitivity from 89.3% to 96.0%, 92.8% to 95.0%, 75.2% to 92.0%, and 93.1% to 97.2% in abnormal eyes, eyes with KC, those with SKC, and normal eyes, respectively. Therefore, the diagnostic accuracy of the AI approach was further improved by including the posterior corneal surface and corneal thickness data. Smadja et al. (2013) applied an MLC to discriminate between normal eyes and KC with 100% sensitivity and 99.5% specificity and between normal and FFKC with 93.6% sensitivity and 97.2% specificity. The MLC showed excellent performance in discriminating between normal eyes and FFKC, thus providing a tool closer to automated medical reasoning. This AI might undoubtedly enable clinicians to detect FFKC before refractive surgery. However, its effect requires further validation since only 372 eyes of 197 patients were included. Similarly, Ruiz Hidalgo et al. (2016) classified 860 eyes into five groups by combining 22 parameters obtained from Pentacam measurements and conducted MLC training. Consequently, the accuracy of the FFKC versus normal task was 93.1%, with 79.1% sensitivity and 97.9% specificity for the FFKC discrimination. Considering the difference between eyes, Kovács et al. (2016) included a “bilateral data” parameter and used a neural network algorithm for modeling. This system on bilateral data of the index of height decentration had a higher accuracy than a single unilateral parameter in differentiating the eyes of all patients with KC from control eyes (area under ROC, 0.96 versus 0.88). Yousefi et al. (2018) developed an unsupervised ML algorithm and applied it to identify and monitor KC stages. Four hundred and twenty corneal topographies, elevations, and pachymetric parameters were also measured. Notably, the specificity of this AI method for identifying normal eyes from those with KC was 94.1%, and the sensitivity for identifying KC in normal eyes was 97.7%. Therefore, this technique can be adopted in corneal clinics and research settings to better diagnose and monitor changes and improve our understanding of corneal changes in KC. Zou et al. (2019) also investigated the diagnosis of healthy corneas, SKC, and KC through ML modeling using Pentacam data of participants in 2018. The diagnostic accuracy of this model for SKC and normal corneas was 95.53% and 96.67%, respectively, and the AUC of the validation set was 99.36%. Conversely, the accuracy of diagnosis of KC and normal corneas was 98.91%, and the AUC of the validation set was 99.82%. The diagnostic accuracy of the model was 95.53%, which was significantly better than the resident’s with 93.55%. Dos Santos et al. (2019) employed a custom-built ultra-high-resolution OCT (UHR-OCT) system to scan 72 and 70 normal and KC eyes, respectively. Overall, 20,160 images were labeled and used for training in a supervised learning approach. A custom neural network architecture, CorneaNet [Figure 3 depicts CorneaNet, created by Dos Santos et al. (2019)], was designed and trained. This study revealed that CorneaNet could segment both normal and KC images with high accuracy (validation accuracy, 99.56%). Interestingly, CorneaNet could detect KC early and, more generally, examine other diseases that change corneal morphology. Issarti et al. (2019) established a stable, low-cost computer-aided diagnosis (CAD) system for early KC detection. CAD combines a custom-made mathematical model, feedforward neural network (FFN), and Grossberg-Runge Kutta architecture to detect and suspect KC clinically. The final diagnostic accuracy was >95% for KCS, mild KC, and moderate KC. The algorithm also provides a 70% reduction in computation time while increasing stability and convergence regarding traditional ML techniques.

Some studies have focused on staging KC severity. Kamiya et al. (2019) applied the DL of color-coded maps, measured using swept-source AS-OCT, to evaluate the diagnostic accuracy of KC. They included a total of 304 eyes [grades 1 (108 eyes), 2 (75 eyes), 3 (42 eyes), and 4 (79 eyes)] according to the Amsler–Krumeich classification and 239 age-matched healthy eyes. This AI system effectively discriminated KC from normal corneas (99.1% accuracy) and further classified the grade of the disease (87.4% accuracy). Two studies used topography images to detect and stage KC (Kamiya et al., 2021a; Chen et al., 2021). Both studies had high overall accuracies [78.5% (Kamiya et al., 2021a), 93% (Chen et al., 2021)], with better performance on color-coded maps than the raw topographic indices. Malyugin et al. (2021) trained an ML model using topography images and visual acuity to classify KC stages based on the Amsler–Krumeich classification system. The model’s overall classification accuracy was 97%, highest for stage 4 KC and lowest for FFKC. Another study trained an ensemble CNN on Pentacam measurements to differentiate between normal eyes and early, moderate, and advanced KC with a staging accuracy of 98.2% (Ghaderi et al., 2021). Other studies have focused on detecting KC progression, though each study had varying definitions of disease progression. The first study trained a CNN model on AS-OCT images, achieving an 84.9% accuracy in discriminating KC with and without progression (Kamiya et al., 2021b). Another study trained an AI model to predict KC progression and the need for corneal crosslinking using tomography maps and patient age with an AUC of 0.814 (Kato et al., 2021).

Lavric and Valentin (2019) proposed a corneal detection algorithm using CNN to analyze and detect KC and obtained an accuracy rate of 99.33%. Kuo B. I et al. (2020) developed a DL algorithm for detecting KC based on a computer-assisted videokeratoscope (TMS-4), Pentacam and Corvis ST. The AI model has high sensitivity and specificity in identifying KC. Abdelmotaal et al. (2020) used a domain-specific CNN to implement DL. The CNN performance was assessed using standard metrics and detailed error analyses, which include network activation maps. Accordingly, the CNN categorized four map-selectable display images, with average accuracies of 0.983 and 0.958 for the training and test sets, respectively. Furthermore, Shi et al. (2020) created an automated classification system that used MLC to distinguish clinically unaffected eyes in patients with KC from a normal population by combining Scheimpflug camera images and UHR-OCT imaging data. Interestingly, this AI model dramatically improved the differentiable power to discriminate between normal eyes and those with SKC (AUC = 0.93). The epithelial features extracted from the OCT images were the most valuable for the discrimination process. Cao et al. (2021) developed a new clinical decision-making system based on ML, automatically detecting SKC with high accuracy, specificity and sensitivity. Mohammadpour et al. (2022) developed a classifier based on AI, which can help detect early keratoconus. Al-Timemy et al. (2021) trained a hybrid-CNN model to identify features and then used it to train an SVM to detect KC. The final AI model had a 97.70% accuracy in differentiating normal from KC eyes and 84.40% in differentiating normal, KCS, and KC based on the merged development subset and independent validation subset. Kundu et al. (2021) established a universal architecture of combining AS-OCT and AI. It achieves an excellent classification of normal and KC. This AI model effectively classified very asymmetric ectasia (VAE) eyes as SKC and FFKC. Tan et al. (2022) developed a novel method based on biomechanical parameters calculated from raw corneal dynamic deformation videos to quickly and accurately diagnose KC using ML (99.6% accuracy). Ahn et al. (2022) developed and validated a novel AI model to determine a diagnosis of KC based on basic ophthalmic examinations, including visual impairment, best-corrected visual acuity, intraocular pressure (IOP), and autokeratometry. Xu et al. (2022) developed a deep learning-derived classifier (KerNet) that is helpful for distinguishing clinically unaffected eyes in patients with asymmetric keratoconus (AKC) from normal eyes.

Other studies have compared AI algorithms to detect KC. Souza et al. (2010) used three algorithms: SVM, multi-layer perceptron, and radial basis function neural networks. Notably, the three selected classifiers performed optimally, with no significant differences between their performance. Cao et al. (2020) found the RF model outperformed other ML algorithms using tomographic and demographic data. Herber et al. (2021) found that the RF model had good accuracy in predicting healthy eyes and various stages of KC. The accuracy was superior to that of the linear discriminant analysis model. Castro-Luna et al. (2021) also found that the RF outperformed the decision tree model (89% accuracy vs. 71%, respectively), while Aatila et al. (2021) found the RF model to have the highest accuracy when compared with other ML models in detecting all classes of KC.

AI has been used to screen potential candidates for refractive surgery besides detecting KC. For example, Xie et al. (2020) established a system centered on the AI model Pentacam InceptionResNetV2 Screening System (PIRSS) to screen normal corneas, suspected irregular corneas, early stage KC, KC, and PRK corneas. The PIRSS system achieved an overall detection accuracy of 95%, similar to that of specialists who were refractive surgeons (92.8%). Recently, Hosoda et al. (2020) have identified KC-susceptibility loci by integrating genome-wide association study (GWAS) with AI, demonstrating that computational techniques combined with GWAS can help identify hidden relationships between disease susceptibility genes and potential susceptibility genes. The above research shows that the AI model is close to an experienced ophthalmologist in the classification and grading of KC.

## 5 AI application in infectious keratitis

Infectious keratitis is one of the most common corneal diseases that significantly causes visual impairment (Papaioannou et al., 2016; Austin et al., 2017; Flaxman et al., 2017; Ung et al., 2019). The disease can be categorized into different types, such as bacterial keratitis (BK) (Tuft et al., 2022), fungal keratitis (FK) (Sharma et al., 2022), herpes simplex virus stromal keratitis (HSK) (Banerjee et al., 2020), or Acanthamoeba keratitis (AK) (de Lacerda and Lira, 2021). Early detection and timely medical intervention of keratitis can prevent the disease progression, thus attaining a better prognosis (Austin et al., 2017; Lin et al., 2019). However, if not diagnosed and treated promptly, keratitis may lead to significant vision loss and corneal perforation (Watson et al., 2018). The diagnosis of infectious keratitis mostly depends on discriminatively identifying the visual features of the infectious lesion in the cornea by a skilled ophthalmologist. AI analysis has been introduced into the field of keratitis diagnosis for automatic real-time identification of abnormal components in corneal images, thereby assisting ophthalmologists in rapidly diagnosing infectious keratitis. Table 3 mainly reviews AI applications for the diagnosis of infectious keratitis.

**TABLE 3:** Summary of studies focused on computer-aided infection keratitis diagnosis.

Year	Authors	Imaging modality	Image size	Databases	AI algorithms	AUC (%)	Accuracy (%)	Sensitivity (%)	Specificity (%)	IoU (%)
2003	Saini et al. (2003)	Clinical data	63	Fungal ulcers/Bacterial ulcers	ANN	—	90.70	76.47/100.00	100.00/76.47	—
2017	Sun et al. (2017)	FSI	48	Corneal ulcers	DCNN	—	86.00 (Dice)	82.00	99.00	—
2018	Wu et al. (2018)	CM	378	Normal and FK	ARBP/SVM	99.01	99.74	100.00	99.45	—
2019	Liu et al. (2020)	CM	1,213	Normal and FK	DCNN/HMF	—	99.95	99.90	100.00	—
2020	Lv et al. (2020)	CM	2088	Normal and FK	ResNet	97.69	93.64	82.56	98.89	—
2020	Kuo M. T et al. (2020)	SLI	288	FK and others	CNN (DenseNet)	65.00	69.40	71.10	68.40	—
2021	Mayya et al. (2021)	SLI	540	FK, BK, HSK, AK, and others	MS-CNN	—	88.96	90.67	87.57	—
2021	Xu F. et al. (2021)	CM	1,089	FK and BK	CNN	98.30	94.20	92.70	95.50	—
2021	Xu Y. et al. (2021)	SLI	115, 408	FK, BK, HSK, and others	CNN/TL	≥92.00	80.00	—	—	—
2021	Li Z. et al. (2021)	SLI	13, 557	Normal, keratitis, and others	DL (DenseNet121)	99.80	98.0	97.70	98.20	—
2021	Hung et al. (2021)	SLI	1,330	FK and BK	CNN (DenseNet161)	85.00	78.60	65.80	87.3	—
2021	Koyama et al. (2021)	SLI/FSI	4,306	FK, BK, HSK, and AK	DL/GBDT	≥94.60	≥90.7	—	—	—
2022	Zhang et al. (2022a)	SLI	4,830	FK, BK, HSK, and AK	CNN	≥86.00	≥70.27	≥70.00	—	—
2022	Ghosh et al. (2022)	SLI	2,167	FK and BK	CNN	90.40	83.00	77.00	—	—

FSI, fluorescein staining image; DCNN, deep convolutional neural network; CM, confocal microscopy; FK, fungal keratitis; ARBP, adaptive robust binary pattern; HMF, histogram matching fusion; SLI, slit-lamp images; BK, bacterial keratitis; HSK, herpes simplex virus stromal keratitis; AK, acanthamoeba keratitis; MS-CNN, multi-scale convolutional neural network.

In 2003, Saini et al. (2003) assessed the usefulness of ANN for classifying infective keratitis. The trained ANN correctly classified all 63 and 39 of 43 corneal ulcers in the training and test sets, respectively. Specificity for bacterial and fungal categories was 76.47% and 100%, respectively. The accuracy of the ANN was 90.7% and was significantly better than that of the ophthalmologist’s predictions (62.8%). These preliminary results suggest that using neural networks to interpret corneal ulcers requires further development. In 2017, Sun et al. (2017) established a new technique to automatically identify corneal ulcer sites using fluorescein staining images based on a CNN that labels each pixel in the staining image as an ulcer or a non-ulcer. The AI method had a mean Dice overlap of 0.86 compared with the manually delineated gold standard. In 2018, Patel et al. (2018) evaluated the variability of corneal ulcer measurements between cornea specialists and reduced clinician-dependent variability using semi-automated segmentation of ulcers from photographs. Wu et al. (2018) classified normal and FK images based on the newly proposed texture analysis method, adaptive robust binary pattern (ARBP), and the SVM, preprocessed abnormal images to enhance targets and employed the line segment detector algorithm to detect hyphae. Interestingly, it could perfectly separate abnormal from normal corneal images with an accuracy of 99.74%. Liu et al. (2020) proposed a new CNN framework for automatically diagnosing FK using data augmentation and image fusion. This study indicated that the accuracy of conventional AlexNet and VGGNet were 99.35% and 99.14%, those of AlexNet and VGGNet based on mean fusion were 99.80% and 99.83%, and those of AlexNet and VGGNet based on histogram matching fusion (HMF) were 99.95% and 99.89%. Additionally, this novel CNN framework perfectly balances diagnostic performance and computational complexity and can improve real-time performance in diagnosing FK.

**FIGURE 4:**
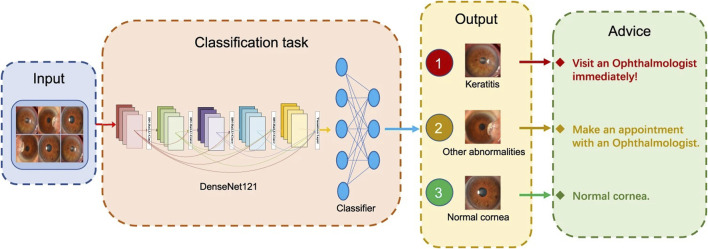
Workflow of the DL system in clinics for detecting abnormal cornea findings (Li Z. et al., 2021).

Lv et al. (2020) developed an AI system based on the DL algorithm for the automated diagnosis of FK in IVCM images. The AI system exhibited satisfactory diagnostic performance (93.64% accuracy) and effectively classified FK in various IVCM images. Xu F. et al. (2021) established an interpretable AI (XAI) system based on Gradient-weighted Class Activation Mapping (Grad-CAM) and Guided Grad-CAM and used IVCM images for FK detection. With better interpretability and explainability, XAI-assistance assistance increased the accuracy (94.2%) and sensitivity (92.7%) of competent and novice ophthalmologists significantly without reducing specificity (95.5%). Two studies used SLI images to detect FK (Kuo M. T et al., 2020; Mayya et al., 2021). The diagnostic rate of FK in one study is 69.40% (Kuo M. T et al., 2020), while that of the other study is 88.96% (Mayya et al., 2021). Xu Y. et al. (2021) designed a sequential-level deep model to discriminate infectious corneal diseases effectively by classifying clinical images based on more than 1,10,000 SLI. The model achieved a diagnostic accuracy of 80%, much better than the 49.27% diagnostic accuracy of 421 ophthalmologists. Furthermore, Li Z. et al. (2021) developed an AI system for the automated classification of keratitis, other corneal abnormalities, and normal corneas based on 6,567 SLI (Figure 4 depicts the workflow of the DL system in clinics, which was created by Li et al.). This AI system showed remarkable performance in cornea images captured by different digital slit-lamp cameras and a smartphone with the super macro mode (all AUCs >0.96). Additionally, the system performed similarly to that of ophthalmologist specialists in classifying keratitis, cornea with other abnormalities, and normal corneas.

Furthermore, Hung et al. (2021) applied different CNN to differentiate between BK and FK using SLI. The DL algorithm achieved an average accuracy of 80.0%. Additionally, the diagnostic accuracy for BK and FK ranged from 79.6% to 95.9% and 26.3% to 65.8%, respectively. Koyama et al. (2021) adopted a DL architecture for facial recognition and applied it to determine the probability score for specific pathogens that cause keratitis. 4,306 SLI were studied, including 312 images from internet publications on keratitis caused by bacteria, fungi, acanthamoeba, and HSV. The developed algorithm had a high overall accuracy; for diagnosis, the accuracy/AUC for AK, BK, FK, and HSK was 97.9%/0.995, 90.7%/0.963, 95.0%/0.975, and 92.3%/0.946, respectively. Zhang et al. (2022a) constructed an early IK-aided diagnosis model (KeratitisNet) based on DL. The accuracy of KeratitisNet for diagnosing BK, FK, AK, and HSK was 70.27%, 77.71%, 83.81%, and 79.31%, and AUC was 0.86, 0.91, 0.96, and 0.98, respectively. Ghosh et al. (2022) found that compared with the single architecture model, the CNN with ensemble learning performs best in distinguishing FK from BK.

In addition to the abovementioned discrimination between different keratitis types, there is also a study of a fully-automatic DL-based algorithm for segmenting ocular structures and microbial keratitis biomarkers on SLI (Loo et al., 2021). Tiwari et al. (2022) trained a CNN to differentiate active corneal ulcers from healed scars from SLI. The AI model was tested on internal (India) and external (the United States) data sets and achieved high performance (AUCs > 0.94). Koo et al. (2021) reported that the model detects hyphae more quickly, conveniently, and consistently through DL using CM images in real-world practice. The performance of this AI model showed high sensitivity and specificity. The above research shows different performances in the diagnosis and classification of different keratitis by AI model, but basically the accuracy is gradually improving.

## 6 AI application in dry eye

Dry eye is one of the most common ocular surface diseases in clinical practice, characterized by a loss of homeostasis of the tear film and accompanied by ocular abnormalities, such as tear film instability and hyperosmolarity, ocular surface inflammation and damage, and neurosensory abnormalities (Craig et al., 2017a; Craig et al., 2017b; Stapleton et al., 2017). As the most common trigger of dry eye (Craig et al., 2017b), MGD is associated with many other ocular diseases (Sullivan et al., 2018; Lekhanont et al., 2019; Llorens-Quintana et al., 2020) and systemic factors (Arita et al., 2019; Sandra Johanna et al., 2019; Wang et al., 2020), which affect patients’ quality of life, causing ocular irritation, ocular surface inflammation, and visual impairment (Sabeti et al., 2020). Therefore, evaluating the function of meibomian glands (MGs) in patients with dry eyes is essential. Furthermore, MG morphology is closely associated with the severity of MGD, and the MG image index indicates their health (Giannaccare et al., 2018). Recently, researchers have started employing image processing and image analysis software such as ImageJ to perform morphological analysis of the structure of MGs. However, semi-quantitative analysis requires manual labeling of each image, which is labor-intensive and inefficient. The efficiency of AI technology in image recognition is much higher than that of manual analysis, and the cost is significantly reduced. Table 4 mainly reviews AI applications for the diagnosis of dry eye.

**TABLE 4:** Summary of studies focused on computer-aided dry eye diagnosis.

Year	Authors	Imaging modality	Image size	Databases	AI algorithms	AUC (%)	Accuracy (%)	Sensitivity (%)	Specificity (%)	IoU (%)
2017	Peteiro-Barral et al. (2017)	Tearscope plus	105	Tear film classification	SVM/MLC	≥92.00	≥94.00	≥84.00	≥96.00	—
2018	Su et al. (2018)	Digital camera	80	Break-up, non-break-up, eyelid, eyelash, and sclera (TBUT)	DCNN	96.00	98.00	83.00	95.00	—
2019	Wang J. et al. (2019)	Keratograph 5M	706	MG trophy area	CNN	—	97.60	—	—	95.40
2020	Maruoka et al. (2020)	CM	137	Normal and obstructive MGD	Ensemble DL	98.10	—	92.10	98.80	—
2020	Stegmann et al. (2020)	OCT	6,658	Tear meniscus segmentation	DCNN	—	≥99.20	≥96.36	≥99.86	≥93.16
2021	Chase et al. (2021)	AS-OCT	27,180	Normal and dry eye	CNN	—	84.62	86.36	82.35	—
2021	Deng et al. (2021)	Keratograph 5M	528	Tear meniscus segmentation	FCNN	—	—	≥84.40	—	82.50
2021	Zhang et al. (2021)	CM	8,311	Normal, obstructive and atrophic MGD	CNN	≥97.30	≥97.30	≥88.80	≥95.40	—
2021	Setu et al. (2021)	Keratograph 5M	728	MG segmentation and morphology assessment	DL/TL	96.00	84.00 (Dice)	81.00	—	—
2021	Wang J. et al. (2021)	Keratograph 5M	1,443	MG segmentation and ghost glands assessment	DL	—	—	84.40	71.70	63.00
2021	Dai et al. (2021)	Keratograph 5M	120	MG morphologic	CNN	—	—	—	—	90.77
2022	Zhang et al. (2022b)	Keratograph 5M	4,006	MG density and meiboscore	Mask R-CNN/TL	90.00	—	88.00	81.00	93.00
2022	Vyas et al. (2022)	TOPCON DV3 camera	30	Normal, break-up, blink, or noise (TBUT)	CNN/TL	80.00	83.00	87.00	89.00	—

TBUT, tear film break-up time; MG, meibomian gland; MGD, meibomian gland dysfunction; FCNN, fully convolutional neural networks. Keratograph 5M: (OCULUS, Arlington, WA), a clinical instrument that uses an infrared light with wavelength 880 nm for MG imaging.

In 2019, Wang J. et al. (2019) established a DL approach to digitally segment the MG atrophy area and compute the percentage atrophy in meibography images. In total, 497 meibography images were used to train and adjust the DL model, while the remaining 209 images were applied for evaluation. The AI algorithm achieves 95.6% meiboscore grading accuracy on average, significantly outperforming the specialist by 16.0% and the clinical team by 40.6%. This study presents an accurate and consistent gland atrophy evaluation method for meibography images based on deep neural networks and may contribute to an improved understanding of MGD. However, this AI system could only predict the MG atrophy region rather than individual MG morphology. In 2020, Maruoka et al. (2020) evaluated the ability of DL models to detect obstructive MGD using *in vivo* confocal microscopy (IVCM) images. For the single DL model, the AUC, sensitivity, and specificity of diagnosing obstructive MGD were 0.966%, 94.2%, and 82.1%, respectively, and for the ensemble DL model, 0.981%, 92.1%, and 98.8%, respectively. Zhang et al. (2021) developed a DL algorithm to check and classify IVCM images of MGD automatically. By optimizing the AI algorithm, the classifier model displayed excellent accuracy. The sensitivity and specificity of the AI model for obstructive MGD were 88.8% and 95.4%, respectively, and for atrophic MGD, 89.4% and 98.4%, respectively. Furthermore, Zhou et al. (2020) used the transfer-learning mask R-CNN to build a model. The model evaluated each image in 0.499 s, whereas the average time for clinicians was more than 10 s. This study also included 2,304 MG images to construct an MG image database. The proportion of MGs marked by the model was 53.24% ± 11.09%, and the artificial marking was 52.13% ± 13.38%. Therefore, this model can improve the accuracy of examinations, save time, and be used for clinical auxiliary diagnosis and screening of diseases related to MGD. Prabhu et al. (2020) proposed an automated algorithm based on DL to segment MGs and evaluated various features for quantifying these glands. This study also analyzed five clinically relevant metrics in detail and found that they represented changes associated with MGD.

**FIGURE 5:**
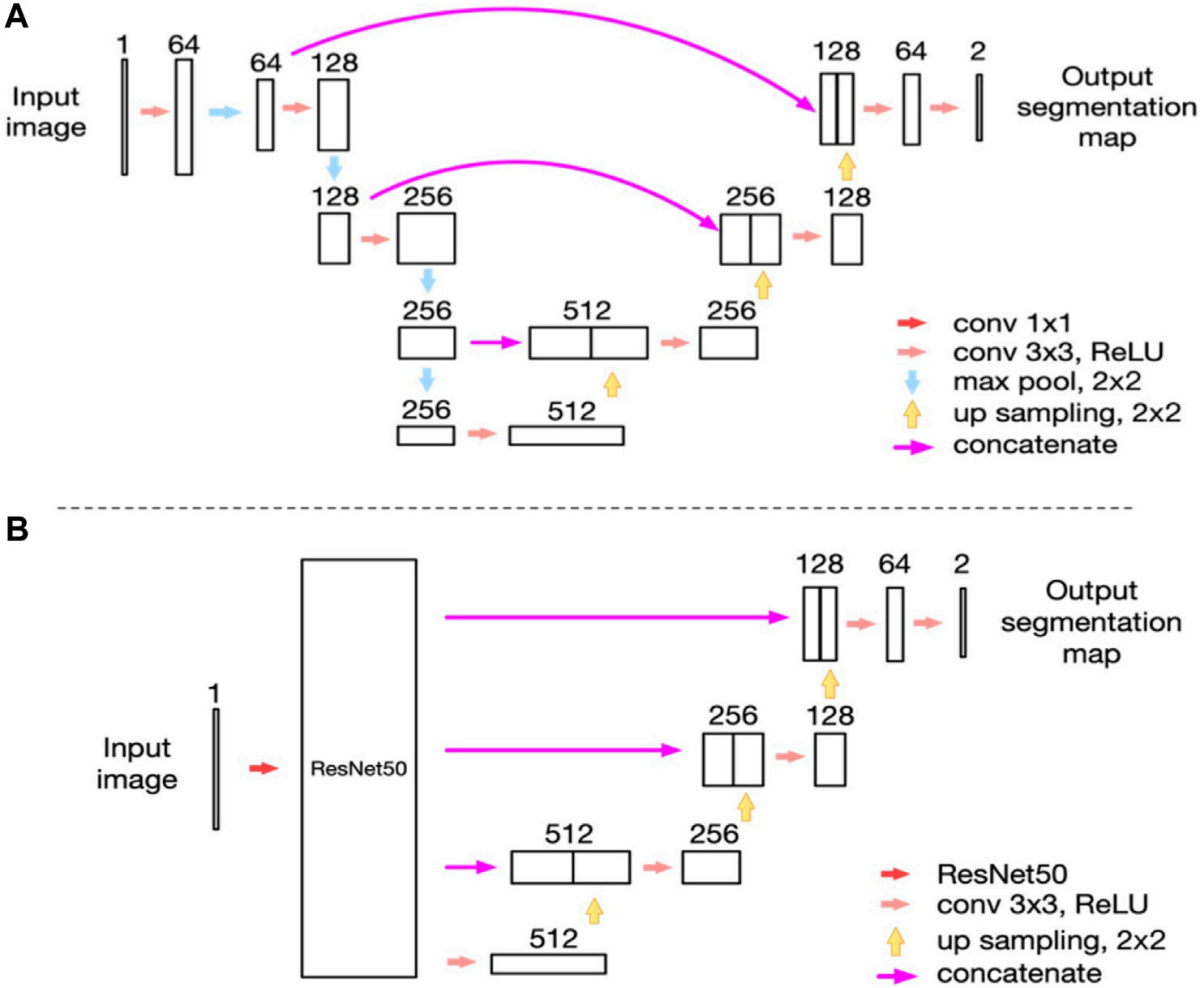
Network structure (Zhang et al., 2022b). **(A)** The network structure of the modified U-net model as we reported previously; **(B)** The network structure of the ResNet50_U-net model in this study.

In 2021, we proposed a novel MGs extraction method based on CNN (Dai et al., 2021) with an enhanced mini U-Net. Consequently, the IoU achieved 0.9077, and repeatability was 100%. The processing time for each image was 100 ms. We identified a significant and linear correlation between MG morphology and clinical parameters using this method. This study provided a new method for quantifying morphological features of MG obtained by meibography. Furthermore, we used an advanced AI system based on ResNet_U-net (Figure 5 depicts the network structure created by Zhang et al.) to assess the effect of MG density in diagnosing MGD (Zhang et al., 2022b). The updated AI system achieved 92% accuracy (IoU) and 100% repeatability in MG segmentation. The AUC was 0.900 for MG density in all eyelids. Sensitivity and specificity were 88% and 81%, respectively, at a cutoff value of 0.275. We compared the correspondence between MG density and meiboscore, as shown in Table 5. Thus, MG density is an effective index for MGD, particularly supported by the AI system, which could replace the meiboscore.

**TABLE 5:** Comparison table of MG density and meiboscore (Zhang et al., 2022b).

	MG density					
	Upper eyelid (1,620)			Lower eyelid (2,386)		
	Median (IQR)	H-value	*P*	Median (IQR)	H-value	*P*
Meiboscore 0	0.30 (0.25–0.33)	882.932	<0.001	0.19 (0.14–0.23)	596.815	<0.001
Meiboscore 1	0.25 (0.21–0.29)			0.17 (0.13–0.21)		
Meiboscore 2	0.15 (0.12–0.18)			0.13 (0.10–0.17)		
Meiboscore 3	0.10 (0.06–0.12)			0.07 (0.04–0.11)		

MG, meibomian gland; IQR, interquartile range.

**FIGURE 6:**
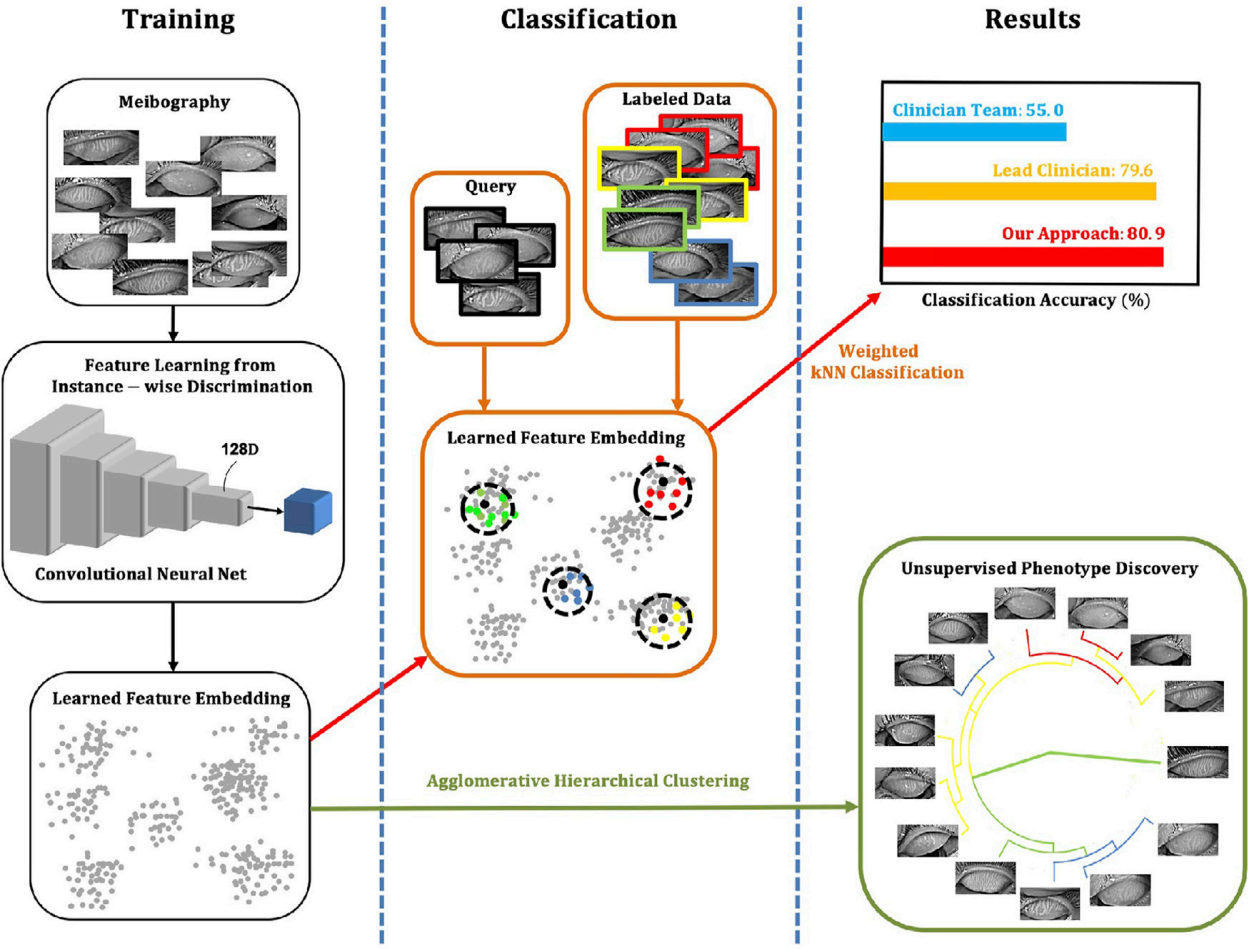
Overview of the approach (Yeh et al., 2021). The NPID is applied to learn a metric by feeding unla-beled meibography images and then to discriminate them according to their visual similarity. This approach measures atrophy severity and discovers subtle relationships between meibogra-phy images. There is no required image labeling, serving as ground truth for training.

In 2021, Khan et al. (2021) established a model based on adversarial learning, a conditional generative adversarial network (C-GAN), to accurately detect, segment, and analyze MG. This technique significantly improved the inability of existing methods to quantify irregularities in infrared images of the MG regions. Additionally, this technique outperformed state-of-the-art results for detecting and analyzing the dropout area of the MGD. Setu et al. (2021) proposed an automatic infrared MG segmentation method based on DL (U-Net). The model was trained and evaluated using 728 anonymized clinical meibography images. The average precision, recall, and F1 scores were 83%, 81%, and 84% on the testing dataset, with an AUC value of 0.96, based on the ROC curve and the Dice coefficient of 84%. Single-image segmentation and morphometric parameter evaluations had an average of 1.33 s. Wang J. et al. (2021) developed an automated AI method to segment individual MG regions in an infrared meibography image and analyzed their morphological features. The AI algorithm, on average, achieved 63% mean IoU in segmenting glands, 84.4% sensitivity and 71.7% specificity in identifying ghost glands. Yeh et al. (2021) established an unsupervised feature learning method based on non-parametric instance discrimination (NPID) to automatically measure MG atrophy (Figure 6 illustrates an overview of the approach created by Yeh et al.). 497 meibography images were used for network learning and tuning, and the remaining 209 images were applied for network model evaluations. The proposed NPID achieved an average 80.9% meiboscore grading accuracy, outperforming the clinical team by 25.9%. Therefore, this method may aid in diagnosing and managing MGD without prior image annotations, which require time and resources.

Dry eye is complicated to diagnose since there is no single characteristic symptom or diagnostic measure. Other studies have employed AI to detect tear film, tear meniscus height (TMH), corneal morphology and blinking to diagnose dry eye besides the abovementioned assessment of dry eye by AI detection of MGs morphology. Diego et al. (Peteiro-Barral et al., 2017) proposed a method that automatically assessed tear film classification and demonstrated its effectiveness. This method applied class binarization and feature selection for optimization purposes. Su et al. (2018) proposed an automatic method to detect the fluorescent tear film break-up area using a CNN model and to define its appearance as CNN-BUT. The sensitivity and specificity of CNN-BUT in screening patients with dry eye were 0.83 and 0.95, respectively. Vyas et al. (2022) proposed a tear film break-up time (TBUT) -based dry eye detection method that detects the presence/absence of dry eye from TBUT video. This AI system exhibits high performance in classifying TBUT frames, detecting dry eye, and severity grading of TBUT video with an accuracy of 83%.

Further, Stegmann et al. (2020) evaluated lower TMH using OCT by automatically segmenting the image data using AI algorithms. The AI segmentation times were approximately two orders of magnitude faster than the previous algorithms. Chase et al. (2021) developed a CNN algorithm to detect dry eye using AS-OCT images with good performance (accuracy = 84.62%, sensitivity = 86.36%, specificity = 82.35%). The epithelial layer and tear film were the learned areas of the AS-OCT images that differentiated images with dry eye from normal. The AI model had a significantly higher accuracy detecting dry eye than corneal staining, conjunctival staining, and Schirmer’s testing. Deng et al. (2021) established a method for the automatic quantitation of lower TMH with FCNN. These neural networks have high performance owing to the modified encoder with a residual block, which has better feature extraction than the original U-Net. Additionally, the overall average IoU for tear meniscus segmentation was 82.5%. Therefore, the algorithm results of the TMH had a higher correlation with the ground truth than manually obtained results. Su et al. (2020) proposed training a deep CNN model to detect superficial punctate keratitis (SPK) automatically, and this AI method can be used to reliably grade the severity of SPK to improve the efficiency (97% accuracy) of dry eye diagnosis. Through AI analysis, Jing et al. (2022) have found a significant correlation between corneal nerve morphological changes in patients with dry eyes and intrinsic corneal aberrations, particularly higher-order aberrations. Zheng et al. (2022) established a blink analysis model using AI to generate a blink profile, which provides a new method for evaluating incomplete blinking and diagnosing dry eye. The above research shows that the AI model has achieved remarkable results in the segmentation of MG morphology in patients with dry eye.

## 7 AI application in other ocular surface diseases

AI has also led to many achievements in the auxiliary diagnosis and treatment of corneal edema, corneal endothelial dystrophy, corneal nerves, corneal epithelial defects, posterior elastic layer detachment, corneal perforation, corneal foreign bodies, and other ocular surface diseases. Veli and Ozcan (2018) established a cost-effective and portable platform based on contact lenses for the non-invasive detection of *Staphylococcus aureus* using a three-dimensional (3D) holographic reconstruction combined with an SVM-based ML algorithm. Interestingly, the method is characterized by low cost and portability, although the study did not include participants for clinical trials. Eleiwa et al. (2020) created and validated a DL model based on VGG19 and transferred learning to diagnose Fuchs endothelial corneal dystrophy. Additionally, Wei et al. (2020) proposed a DL model for automated sub-basal corneal nerve fiber segmentation and evaluation using IVCM). The model achieved an AUC, sensitivity, and specificity of 0.96, 96%, and 75%, respectively. However, this AI model had limitations in that it was not externally validated and could consider all parameters in the IVCM images. Zéboulon et al. (2021) established and verified a novel automated tool for detecting and visualizing corneal edema using OCT. This study trained a CNN to classify each pixel in the corneal OCT images as “normal” or “edema” and to generate colored heat maps of the result. Additionally, the optimal threshold for differentiating normal from edematous corneas was 6.8%, with an accuracy, sensitivity, and specificity of 98.7%, 96.4%, and 100%, respectively. However, the AI model could not quantitatively analyze the severity of edema, and the principle of the model training process output results remains invisible. Li D. F et al. (2021) developed an image analysis system for AS-OCT examination results based on DL technology and evaluated its influence on identifying various corneal pathologies and quantified indices. Furthermore, the labeled AS‐OCT images were used to train corneal pathology detection and stratification models based on the deep CNN algorithm. Interestingly, the average sensitivity and specificity of the corneal pathology detection model were 96.5% and 96.1%, compared with the results of manual labeling. Additionally, the average Dice coefficients of the corneal stratification model for the corneal epithelium and stroma were 0.985 and 0.917, respectively. Deshmukh et al. (2021) developed an automated segmentation DL algorithm for corneal stromal deposits in patients with corneal stromal dystrophy. Segmentation on corneal deposits was accurate *via* the DL algorithm in the well-controlled dataset and showed reasonable performance in a real-world setting. Yoo et al. (2021) developed an AI model to detect conjunctival melanoma using a digital imaging device such as smartphone camera. It showed an accuracy of 94.0% using 3D melanoma phantom images captured using a smartphone camera.

## 8 Discussion

With the development of modern society and the economy, people’s health awareness is gradually improving, and the pressure on ophthalmologists to diagnose and treat will increase. However, although over 2,00,000 ophthalmologists exist worldwide, there is currently a severe shortfall in developing countries (Resnikoff et al., 2012). Furthermore, the number of ophthalmologists is declining in 12% of low-income countries with the lowest ophthalmologist densities and highest population growth rates (Resnikoff et al., 2020). The timely emergence of AI has given rise to optimism in the field of ophthalmology, particularly in areas involving big data and image-based analysis. DL is a branch of ML that employs multi-layer neurons with high-dimensional non-linear transformations in performing high-dimensional data abstraction to extract hidden features (Lecun et al., 2015). Therefore, with the help of DL, we can input many images as samples to the computer and allow the computer to automatically learn the high-dimensional features of the images to determine the intrinsic relationship between the images and the results. DL establishes an intrinsic relationship between input and output through multi-layer CNN mapping, similar to the human learning process. Thus far, various AI models have been developed, such as CNN, deep neural networks, deep belief networks, and RNN. These models have been applied in computer vision, speech recognition, natural language processing, audio recognition, and bioinformatics with excellent results (Lecun et al., 1998; Taigman et al., 2014; He et al., 2016). Additionally, using DL to process and analyze images of ocular surface diseases can significantly improve accuracy and efficiency, reduce manual analysis costs, and overcome errors between different experienced annotators. Currently, different AI models are used for AI applications for different ocular surface diseases. Among them, CNN model accounts for the majority of the AI applications for pterygium, keratitis and dry eye, while RF model has good accuracy in predicting healthy eyes and KC in all stages in the AI application for KC.

DL established a method for computers to automatically learn the hidden features in images and integrate feature learning into building models, thereby reducing the incompleteness caused by artificially designed features. Patterns that are invisible to the naked eye can be picked out. For example, Kermany et al. (2018) trained a DL system to identify retinal OCT images of patients. Surprisingly, the system also accurately identified several other characteristics, including risk factors for heart disease, age, and sex. No one had previously noticed sex variations in the human retina. However, we cannot fully understand its feature extraction logic, leading to the AI “black box” since the DL neural network is very complex and has poor interpretability challenges (Ahuja and Halperin, 2019). Therefore, Kermany et al. (2018) used “occlusion testing” in their study of AI recognition of OCT retinopathy images to study the logic of AI diagnosis. This involved occluding different parts of OCT images of the fundus of patients with retinopathy. The AI erroneously categorized the lesion image as normal after considering the features of a specific section, implying that these features are the basis for the AI’s judgment. Similarly, in analyzing ocular surface diseases using DL models, we can also use occlusion testing to learn the judgment basis of AI to discover new morphological evaluation indicators of ocular surface diseases. An ophthalmic multi-modal diagnostic platform using multiple modules for targeted examination of target tissues has been established and applied clinically. With advances in technology, it may be possible in the future to acquire global three-dimensional data of the eye simultaneously. Correct reading, analysis and diagnosis of acquired data require a more comprehensive and in-depth knowledge base. Compared with human beings, AI has absolute superiority in integrating information, processing data, diagnosis speed, etc.

At present, AI still has certain limitations. 1) Most ML methods have insufficient training and validation sets; therefore, more image data training is needed to improve accuracy, sensitivity, and specificity further. 2) The inspection equipment used by different countries, regions, and medical institutions differ, as do the images obtained by different inspection equipment regarding color and resolution, which will inevitably affect image acquisition and diagnostic accuracies. 3) Current ML methods cannot explain disease diagnosis, of which the output results are learned only from the training set. 4) AI cannot learn effectively for some difficult and rare ocular surface diseases with insufficient data. Therefore, it is difficult to obtain an effective and correct diagnosis rate. Although AI still faces certain challenges in model building, it can assist doctors with objective clinical decisions and lay the foundation for the accurate treatment of patients. These issues must be adequately addressed before AI can be translated into clinical applications in ophthalmology.

In conclusion, AI has great potential to improve the diagnostic efficiency of ocular surface diseases. The novelty of this study is evidenced by its contribution to the existing literature, as it is one of the studies to provide information on research hotspots and trends in the application of AI in diagnosing ocular surface diseases. Furthermore, the results reveal that although AI still faces certain challenges in model building, it can assist doctors with objective clinical decisions and lay the foundation for the accurate treatment of patients. Ultimately, AI algorithms and tools in development for o ocular surface disease are helping us to understand disease pathogenesis, identify disease biomarkers, and develop novel treatments for ocular surface disease.
